# Multi-Dimensional Underwater Point Cloud Detection Based on Deep Learning

**DOI:** 10.3390/s21030884

**Published:** 2021-01-28

**Authors:** Chia-Ming Tsai, Yi-Horng Lai, Yung-Da Sun, Yu-Jen Chung, Mark Shortis

**Affiliations:** 1 Department of Mechanical and Electro-Mechanical Engineering, National Sun Yat-sen University, Kaohsiung 804, Taiwan; d073020009@nsysu.edu.tw (C.-M.T.); lai81.tom@g-mail.nsysu.edu.tw (Y.-H.L.); 2 Naval Meteorological and Oceanographic Office R.O.C., Kaohsiung 804, Taiwan; mrbig.g9114072005@gmail.com; 3 Naval Academy R.O.C., Kaohsiung 804, Taiwan; chungyj@cna.edu.tw

**Keywords:** BV5000, deep learning, underwater point cloud, underwater object detection

## Abstract

Numerous sensors can obtain images or point cloud data on land, however, the rapid attenuation of electromagnetic signals and the lack of light in water have been observed to restrict sensing functions. This study expands the utilization of two- and three-dimensional detection technologies in underwater applications to detect abandoned tires. A three-dimensional acoustic sensor, the BV5000, is used in this study to collect underwater point cloud data. Some pre-processing steps are proposed to remove noise and the seabed from raw data. Point clouds are then processed to obtain two data types: a 2D image and a 3D point cloud. Deep learning methods with different dimensions are used to train the models. In the two-dimensional method, the point cloud is transferred into a bird’s eye view image. The Faster R-CNN and YOLOv3 network architectures are used to detect tires. Meanwhile, in the three-dimensional method, the point cloud associated with a tire is cut out from the raw data and is used as training data. The PointNet and PointConv network architectures are then used for tire classification. The results show that both approaches provide good accuracy.

## 1. Introduction

Object detection on roads has been observed to be a recurring theme in self-driving cars. Several sensors can be used to obtain information regarding object(s) on a road. For vision-based detection, the two-dimensional (2D) information obtained from a camera can be used to detect cars, pedestrians, and motorcycles, among other objects. Numerous methods, such as Faster-RCNN [1], SSD [2], and YOLOv3 [3], have been popularly used in recent years. These network architectures exhibit high accuracy with regard to many image features. However, the lack of distance information provided by cameras has led another type of sensor to gain popularity. This sensor involves a three-dimensional (3D) light detection and ranging (Lidar) technique. A 3D Lidar will emit a laser beam and measure the distance by calculating the time of flight and angle of the returning light signal. Several laser beams can be lined up and rapidly rotated to obtain numerous points around the sensor. Many points can thus be gathered and these are referred to as a point cloud. The 3D Lidar can generate a 3D perspective of its surroundings. The ModelNet40 dataset has been used to classify an object from pure point cloud data [4,5]. This dataset is composed of a significant amount of offline computer-aided design (CAD) data and includes 40 types of objects. 3D Lidar point cloud information was used in a separate study to detect an object’s location on a road and to classify its category [6]. These studies all demonstrated land applications. In addition to those conducted on land, several researchers have expanded their studies to underwater applications. For example, [7] discussed many types of optical and acoustic sensors that could be utilized in underwater archeology.

In water, electromagnetic signals rapidly decay and light cannot reach far distances if the water is turbid or very deep (e.g., the deep sea). Hence, both optical and electromagnetic waves are unsuitable for underwater applications under certain conditions. Fortunately, acoustic signals propagate well through a water medium and are exempt from the issues mentioned above. Sensors such as forward-scan sonar, side-scan sonar, and multi-beam sonar are commonly used in marine applications. For example, an unsupervised, statistically based algorithm was proposed to detect underwater objects [8]. A higher-order statistics representation of the synthetic aperture sonar image was used to detect highlights and form a region-of-interest (ROI). A support vector machine (SVM) was then used to classify the ROI results. As a second example, a synthetic aperture sonar image can also be used to detect underwater unexploded ordnance [9] by transferring convolutional neural networks. The result represented that if the data was limited, the performance of transfer learning was better than that of the training convolutional neural networks (CNNs) from scratch. In [10], a CNN has been proven effective in extracting features from an image. A multi-scale and multi-column CNN was used to detect sonar images. A novel transfer learning method based on progressive fine-tuning was used to accelerate the model’s training. In a separate study [11], an end-to-end underwater object detection method was proposed with forward-scan sonar image. This method can also be used to detect the unlabeled and untrained object and get robust result. In [12], a deep learning method was proposed to detect and remove the crosstalk noise from a forward-scan sonar image. This method can be used to generate a more accurate point cloud. Existing studies have conducted significant research on 2D data obtained from sonar images. In some studies, 3D data were generated from 2D data [13,14]. Furthermore, in another study, 3D data were directly obtained from a sonar sensor. This sensor is the Blueview BV5000 3D mechanical scanning sonar (MSS) designed by Teledyne Technologies (Thousand Oaks, CA, U.S.). Traditional photography imaging techniques cannot detect damage in a structure underwater, but the BV5000 can replace other types of sensors to inspect a bridge pier for damage [15]. Moisan et al. [16] employed the BV5000 sonar to scan both sides of an underwater channel, while using a laser to obtain the shore wall above the water. The data from the two types of sensors were aligned to construct a full 3D reference model of a canal tunnel. A BV5000 precision test was also conducted in [17]. The accuracy of the laser data was measured in centimeters so that it could be used as a reference with which to compare the BV5000 data. The experimental results indicated that the BV5000 could detect gross defects such as stone cracks or cavities present in a structure. Terrestrial laser scanning and 3D Lidar on land have similar applications to the BV5000, but cannot work effectively underwater.

Although the abovementioned studies have conducted significant research regarding underwater environments, they did not perform a multi-scale comparison based on 2D and 3D aspects. The present study uses the BV5000 to acquire 3D point cloud data underwater. The data first undergoes pre-processing to remove redundant point clouds. The goal is to find anti-collision tires lying on the seabed using two types of dimensional studies. With respect to the 2D aspect, the point cloud data are compressed from 3D to 2D and thus provide a bird’s eye view image of the point cloud. The neural network architectures, Faster R-CNN and YOLOv3, are then used for tire detection. For the 3D aspect, the point clouds of the tires are cut out from the raw data and transformed into the ModelNet40 [18] data format to provide a training dataset. The 3D convolutional neural network architectures, PointNet [4] and PointConv [19], are applied to obtain 3D classification results. The results demonstrate the accuracy of the models in both dimensions. 

Section 2 presents a comparison of the characteristics of the BV5000 sensor with those of other acoustic sonars. Section 3 provides a description of the steps taken to obtain the data and describes the pre-processing steps for raw point clouds. The 2D and 3D neural network structures are applied to the data for the different dimensions. Section 4 presents the accuracy results obtained from applying the different approaches. The experimental results demonstrate that a single datum can be expressed in multiple dimensions. Section 5 discusses the conclusions of this study.

## 2. Acoustic Sonar Sensors

Sound navigation and ranging is a technique that uses electric equipment to accomplish detection and communication missions underwater by utilizing the propagation characteristics of sound waves. In water, the speed of sound is mainly affected by temperature. The sound wave can be used to calculate the time of flight to measure the distance from the sensor to the seabed or objects. Currently, sonars have become indispensable equipment for merchant ships, fishing boats, and military ships. Sonar can be divided into active and passive types. Active sonar will transmit sound waves and receive echoes to detect a target, while passive sonar only receives sound waves from surrounding objects. Active sonar can be used to detect mines, create noise, or perform underwater exploration. Passive sonar is widely used in submarine detection applications. This section introduces some common active acoustic sonar technologies, including side-scan sonar, multi-beam echo sounder, and the BV5000. The advantages and disadvantages are discussed, explaining the reasons for choosing the BV5000 as the sensor to be used in this study.

### 2.1. Side-Scan Sonar

A side-scan sonar consists of four parts: a navigation system that receives the positioning information from a global positioning system (GPS), a “tow fish” that consists of a soundwave transmitter and receiver, a central computer that digitizes sonar echo signals, records data, and receives positioning data, and finally a control unit that transmits control commands to the tow fish and returns gathered echo signals to the computer. Furthermore, data processing software is used to instantly display underwater images.

The side-scan sonar is usually towed behind a boat. The fan-shaped sound waves are launched from the sound drums on both sides of the tow fish. The uneven seabed and unique seabed substrate produce different forms of scattered energy. Strong and weak signals can be represented by different color shades and are displayed as a 2D grayscale image. Side-scan sonar can be used for large-scale exploration of the seabed geography or to perform target searches in water [20,21].

### 2.2. Multi-Beam Echo Sounder

The multi-beam echo sounder is essentially a combination of many sonar devices emitting multiple fan-shaped sound waves. It can capture hundreds of depth scans at a time to form a fully covered water depth band, build a high-precision map of the sea floor [22], and obtain the depth of the seabed. The echo sounder greatly improves the efficiency of seabed terrain detection. Additionally, it can be used to perform submarine substrate classification based on the echo intensities of sound waves reflected by different materials [23,24]. It has wide applications in marine resource exploration, gas hydrate detection, and other types of large-scale exploration.

### 2.3. Blueview BV5000 3D MSS

The BV5000 is a new 3D mechanical scanning sonar (MSS) that can provide 3D laser-like scanning properties underwater and create high-resolution 3D point cloud scenes of an underwater area, structure, and objects. It can operate under low and zero visibility conditions and can be integrated with traditional laser scan imagery. It is lightweight and can be deployed on a tripod or on a remotely operated underwater vehicle (ROV). Its mechanical design enables the generation of both fanwise and spherical scan data. In one study [25], an ROV and a BV5000 were used to construct a 3D map of an underwater shipwreck. It can also be applied to bridge inspections [15], oil rig decommissioning, and other underwater 3D surveys. 

Table 1 compares the various sensors. The BV5000 was chosen to collect data for several reasons. Compared with the side-scan sonar, the BV5000 does not require information on the vehicle’s speed and distance from the sea floor. Compared with the multi-beam echo sounder, the BV5000 can be used in shallow water and does not require additional equipment such as GPS, an inertial measurement unit (IMU), or a vehicle to transport it. It can obtain high-resolution data and is simple to use. The operator only needs to mount it on a tripod and submerge the tripod into the water, set some parameters, and press the start button. The BV5000 will start collecting the surrounding data. The BV5000 was chosen for data collection because of these advantages.

## 3. Methods

**Figure 1:**
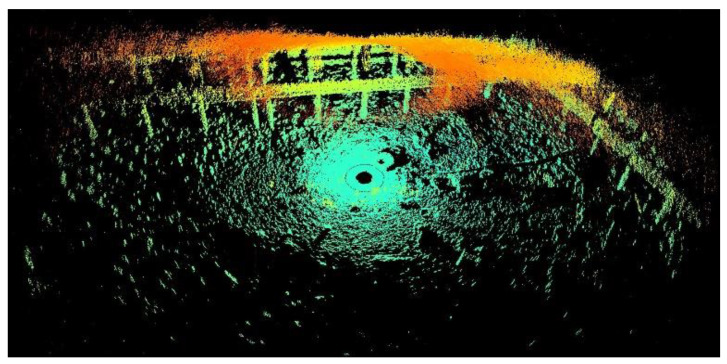
Raw data obtained from the BV5000.

This study aimed to locate abandoned tires and investigate the data from 2D and 3D aspects. The BV5000 was used to obtain a 3D point cloud underwater. It was mounted on a tripod and then vertically placed in water. It then gradually sank and settled on the seabed. The operator pressed a button to initiate data collection. The sound-emission vertical beam angles were −45°, −15°, 15°, and 45°, which were set by the manufacturer. The data obtained using these four angles were spliced when the mission was completed. The results obtained by the BV5000 are depicted in Figure 1. 

**Figure 2:**
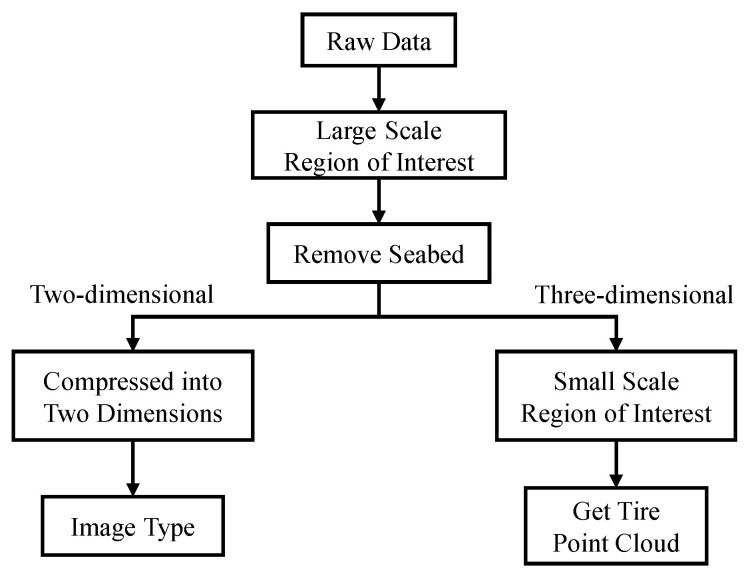
Data pre-processing flow chart.

Figure 1 depicts an image of a fully scanned underwater environment containing several underwater objects. This data is composed of many points, called point cloud in this paper. The location of the BV5000 is the origin of coordinates from the point cloud. Every point records the relative coordinates from the BV5000. The colors indicate different heights from different points to the BV5000. The density of point clouds becomes sparse as the distance to the BV5000 increases. These incomplete point clouds make it difficult to generate actual objects and increase the computation time. To reduce data complexity, two similar pre-processing steps must be performed in both 2D and 3D. Figure 2 outlines these data pre-processing steps. The purpose of these steps is to obtain 2D image data and 3D tire point cloud data. A large-scale region of interest was initially chosen to substantially remove boundary noise. Subsequently, a random sample consensus (RANSAC) algorithm was used to remove the seabed. This step ensured that the tires became more visible. After removing the seabed, the 2D and 3D aspects were pre-processed separately. Regarding the 2D aspect, the point cloud without the seabed was compressed into an image by setting the height value of all points to zero. The output data therefore appeared to present a bird’s eye view of the underwater environment. Regarding the 3D aspect, another more precise region of interest was set to cut out each tire from the entire point cloud. The 3D point cloud of each tire was converted to the Modelnet40 format. Finally, Faster R-CNN and YOLOv3 were used to train the models to detect tires in 2D data, while PointNet and PointConv were used for 3D data.

### 3.1. Choosing the Region of Interest

The BV5000 has a measurement range of up to approximately 30 m in terms of radius, while tires measure only up to 50 cm in size. Most of the point clouds obtained at a large distance were sparse and consisted of insignificant points that could resemble an object. Therefore, choosing a practical region of interest is the first step, as it can substantially reduce the amount of data to be processed. The length and width of the ROI were set to 15 m each, which was decided by the user. If the object was big enough to form a shape at far distance, the range could be increased. The height depended on whether the BV5000 was settled on highland or lowland in underwater. If the BV5000 was settled on highland, the height could increase and vice versa. This setting could substantially remove redundant points above the seabed. 

### 3.2. Removing the Seabed

After pre-processing to remove the boundary point cloud beyond 15 m, the objects were still not sufficiently clear to be distinguished in the raw data. The seabed was rugged because of different substrate materials and soil deposition, requiring a method to remove the seabed from the data. For land conditions, 3D Lidar can obtain 3D point cloud data on the ground. Typically, the ground is flat and can be removed by setting a height filter. However, the uneven seabed could not be directly removed by imitating the method employed on land. To address this issue, a random sample consensus (RANSAC) [26] algorithm was used to remove the seabed. Using this method, a plane was identified to approximate the seabed and separate it from the raw point cloud. The tires became visible after this processing step.

### 3.3. Random Sample Consensus (RANSAC) Algorithm

The RANSAC algorithm was first proposed in 1981 [26]. It has wide applications in the field of computer vision and mathematics. The basic assumption of the RANSAC algorithm is that correct (inliers) and abnormal (outliers) data exist, which means noise data exists. The RANSAC algorithm assumes that if a correct set of data is given, the model parameters can be calculated to fit these data. The RANSAC concept includes two steps, assumed geometry model and iteration, which are presented as follows:

(1) The seabed is assumed to be flat although in the real world the seabed is uneven; hence, the normal vector was set as (0,0,1) for the *z*-axis to fit a plane to the point cloud under additional orientation constraints.
(2) The seabed thickness was set to 20 cm in this study. This represents the maximum distance allowed from an inlier point to the plane. However, the thickness may be changed for different experimental locations.
(3) Points falling within the distance of 20 cm were found.

Because only a few objects were on the seabed, most data points belonged to the seabed. The steps mentioned above can effectively find a plane to fit the seabed. The following steps can remove the seabed from the data to obtain a clear point cloud:

(4) The inlier points considered part of the seabed were removed.
(5) A point cloud without the seabed was thus obtained.

### 3.4. Pre-Processing and Expansion of 2D Data

This study investigated two types of dimensional data. To obtain 2D data, the 3D point cloud data without seabed were transformed to a 2D image by setting the height value of all points to zero. This process can detect tires from the bird’s eye view. The image was rotated in 5° increments to expand the data quantity to provide data augmentation for training of the deep learning neural networks.

### 3.5. Pre-Processing and Expansion of 3D Data

**Figure 3:**
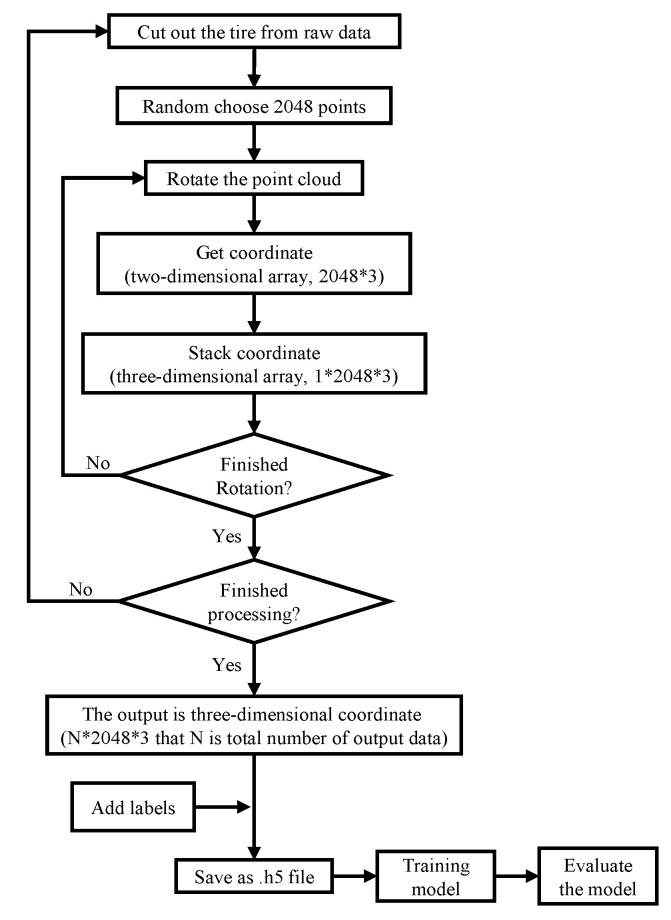
Data augmentation flow chart.

**Figure 4:**
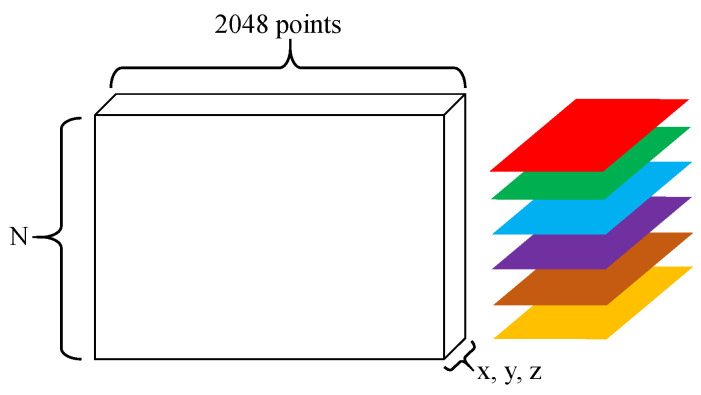
Schematic diagram of the data stack.

Although the point cloud without a seabed made a tire more clearly visible, other miscellaneous point clouds were still present. To obtain a precise point cloud of a tire, a small-scale ROI was defined to extract the entire tire from the removed seabed data. Data augmentation was used to expand the data quantity because the point cloud data representing tires were insuffection for deep learning purposes. Figure 3 depicts the process for increasing the quantity of data after the point clouds were extracted. The steps for expansion are as follows:

(1) The data for each tire consisted of many points, and therefore, 2048 points were randomly selected from these points first.
(2) The point cloud coordinates of a tire can be considered as a 2D array. One dimension is comprised of 2048 points, while the other dimension is based on x, y, and z coordinates (as shown in Figure 4).
(3) The 2048 points were rotated in 5° increments to change their coordinates.
(4) The 2D array can be stacked into a 3D array, where N is the third dimension, which denotes the number of data samples (as shown in Figure 4).
(5) Determine whether the rotation procedure is complete. If not, return to step (3) to rotate the data again.
(6) Determine whether all processing is complete. If not, return to step (1) to read new data.
(7) The data are exported into ModelNet40 format.
(8) Use PointNet and PointConv to train the models.
(9) Evaluate the models’ accuracy.

The pre-processing steps mentioned above substantially reduced the amount of redundant data. The resulting data were then converted into image and point cloud types. The former was used to train the 2D models Faster R-CNN and YOLOv3. The latter type was used to train the 3D models PointNet and PointConv.

### 3.6. Combining 3D Data with ModelNet40

By the data augmentation technique, one data point can be expanded into 72 datapoints. ModelNet40 included 40 kinds of point cloud objects like bottle, door or sofa, etc. 2048 points were randomly chosen from ModelNet40 data and tire data. They were transformed to hdf5 format. The training data were 9840 for ModelNet40 and 864 for tire while the testing data were 2468 for ModelNet40 and 432 for tires. These two kinds of dataset were mixed to train the models. The models are proved to be excellent if they can well classify these 41 kinds of objects.

### 3.7. Faster R-CNN

**Figure 5:**
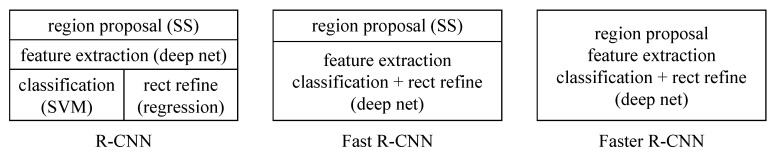
Evolution from R-CNN to Faster R-CNN.

The original recurrent-CNN (R-CNN) neural network architecture was proposed in 2014 [27], and later evolved into Fast R-CNN [28] and Faster R-CNN [1]. Faster R-CNN neural networks are widely used in many computer vision applications [29,30,31] and have the advantage of high-accuracy detection results. The evolution to Faster R-CNN is shown in Figure 5. Thus, the traditional selective search method for extracting targets was replaced by an improved training network. Faster R-CNN uses neural networks to combine four modules: region proposal, feature extraction, classification, and bounding box regression to train an end-to-end network. The development of these networks greatly improved the processing speeds for detection and classification of objects. 

Faster R-CNN includes four basic structures (modules), which are as follows:

(1) Feature extraction network: Serial convolution, rectified linear units, and pooling layers obtain a feature map of the original image.
(2) Region Proposal Network (RPN): An RPN obtains the approximate location of objects on the feature map and generates region proposals. A softmax layer decides whether the anchors are positive or negative (i.e., if there is an object in the proposed region). The bounding box regression fixes the anchors and generates precise proposals.
(3) ROI Pooling: Evolved from spatial pyramid pooling [32]. Feature maps and proposal information are used to extract proposal feature maps. A fully connected layer determines the target category.
(4) Classification: The proposal feature maps determine the category of a proposal. Bounding box regression is used to obtain the precise locations of the detection boxes.

The anchors mentioned in RPN are a group of bounding boxes having different aspect ratios. This common multi-scale method is used in several detection applications. The anchors often overlap, and therefore, the non-maximum suppression method is applied to find the best anchor. 

All candidate bounding boxes were sorted by score, and the bounding box with the highest score was selected in this study. Other bounding boxes, which overlapped the area with the selected bounding box but exceeded a given threshold, were removed. This leads to a substantial decrease in the number of bounding boxes. Bounding box regression was then applied to fine-tune the positive anchors. This fine-tuning step applied translation and scaling processes to ensure that the proposals were close to the ground truth. 

In Faster R-CNN, convolution layers are used to extract feature maps, as in the case of other detection networks. The loss function for an image is defined as follows:

(1) $$L\left(\left\{p_{i}\right\},\left\{t_{i}\right\}\right)=\frac{1}{N_{cls}}\sum^{\;}L_{cls}\left(p_{i},p_{i}^{*}\right)+\lambda \;\frac{1}{N_{reg}}\sum^{\;}p_{i}^{*}L_{reg}\left(t_{i},t_{i}^{*}\right)$$

In this equation:

(2) $$L_{cls}\left(P_{i},P_{i}^{*}\right)=-\log\left[P_{i}^{*}P_{i}+\left(1-P_{i}^{*}\right)\left(1-P_{i}\right)\right]$$

(3) $$L_{reg}\left(t_{i},t_{i}^{*}\right)=R\left(t_{i}-t_{i}^{*}\right)=smooth_{L1}\left(t_{i}-t_{i}^{*}\right)$$

The entire loss function consists of a class loss part and a regression loss part. In (1), i is the anchor index, and $p_{i}$ is the predicted probability that anchor i is an object. The ground truth label $p_{i}^{*}$ is either 1 or 0, indicating whether the anchor is positive or negative, respectively.$\;t_{i}$ is the prediction coordinate of the bounding box, and $t_{i}^{*}$ is the ground truth box of the positive anchor. $N_{cls}$ is the mini-batch size, $N_{reg}$ indicates the number of anchor locations, λ is a balancing parameter that ensures that the two loss functions have equal weight,$\;L_{cls}$ in (2) is the log loss over two classes (is an object, or not), and $L_{reg}$ in (3) is the regression loss, where R is the loss function (smooth_L1_). The smooth_L1_ parameter, which is defined in [28], is used for the regression parameterization of the four coordinates and can be expressed as follows:

(4) $$smooth_{L1}\left(x\right)=\{\begin{array}{c} 0.5x^{2}\;if\;\left|x\right|<1\\ \left|x\right|-0.5\;otherwise\; \end{array}$$

### 3.8. YOLOv3

YOLOv3 [3] evolved from YOLOv1 [33] and YOLOv2 [34]. High speed and accuracy make it a popular method for vision applications [35,36,37]. The basic idea behind the YOLO algorithm is as follows. First, a feature extraction network is used to extract a feature from an input image to obtain a feature map of size S × S. Second, the input image is divided into an S × S grid cell. If the center of the ground truth object is located in one grid cell, that grid cell is used to predict the object. In YOLOv3, every grid cell will generate three bounding boxes. The bounding box with the maximum intersection over union (IOU) with respect to the ground truth is used to predict the object. The method for predicting the coordinates of the bounding box is based on the previous YOLOv2 method. The formulas used to predict the coordinates are as follows:

(5) $$b_{x}=\sigma \left(t_{x}\right)+c_{x}$$

(6) $$b_{y}=\sigma \left(t_{y}\right)+c_{y}$$

(7) $$b_{w}=p_{w}e^{t_{w}}$$

(8) $$b_{h}=p_{h}e^{t_{h}}$$

(9) $$Pr\left(object\right)*IOU\left(b,object\right)=\sigma \left(t_{o}\right)$$

The parameters $t_{x}$, $\;t_{y}$, $\;t_{w}$, and $t_{h}$ are the outputs of the model prediction. $c_{x}$ and $c_{y}$ are the offsets of the image measured from the top left. $p_{w}$ and $p_{h}$ are the bounding box sizes before the prediction, while $b_{x}$, $b_{y}$, $b_{w}$, and $b_{h}$ are the bounding box center coordinates and size after prediction, respectively. $\sigma \left(t_{o}\right)$ is the class-specific confidence score. The loss function of YOLOv3 is as follows:

(10) $$\begin{array}{c} \lambda_{coord}\overset{S^{2}}{\underset{i=0}{\sum }}\overset{B}{\underset{j=0}{\sum }}1_{i,j}^{obj}\left[{\left(t_{x}-{\hat{t}}_{x}\right)}^{2}+{\left(t_{y}-{\hat{t}}_{y}\right)}^{2}+{\left(t_{w}-{\hat{t}}_{w}\right)}^{2}+{\left(t_{h}-{\hat{t}}_{h}\right)}^{2}\right]\\ +\overset{S^{2}}{\underset{i=0}{\sum }}\overset{B}{\underset{j=0}{\sum }}1_{i,j}^{obj}\left[-\log\left(\sigma \left(t_{0}\right)\right)+\overset{C}{\underset{k=1}{\sum }}BCE\left({\hat{y}}_{k},\sigma \left(S_{k}\right)\right)\right]\\ +\lambda_{noobj}\sum_{i=0}^{S^{2}}\sum_{j=0}^{B}1_{i,j}^{noobj}\left[-\log\left(1-\left(t_{0}\right)\right)\right] \end{array}$$

The loss function consists of the bounding box offset prediction loss and the confidence prediction loss. The location and size losses use the sum squared error; class and confidence losses use binary cross entropy. ${\hat{t}}_{*}$ is the ground truth, and $t_{*}$ is the prediction truth. *S* is the number of grid cells. *B* is the bounding box number. $1_{i,j}^{obj}$ denotes whether the jth bounding box prediction in cell i is responsible or not. BCE is the binary cross entropy. Because many grid cells do not contain any object in an image, $\lambda_{coord}$ and $\lambda_{noobj}$ are used to prevent this condition from impacting the model training.

The first improvement involving YOLOv3 is the use of multiple scales to make predictions. The number of bounding boxes is much greater than that of YOLOv2. This method improves the detection accuracy with respect to small targets. The second improvement is multi-label classification. The object classification layer uses logistics instead of softmax to support multi-label objects. Each candidate box can predict multiple classifications. The third improvement is the network architecture. YOLOv3 comprises 53 layers, while YOLOv2 consists of 19 layers. YOLOVv3 uses the residual network method from ResNet [38] to resolve the degenerate problem associated with a deep network and creates a deeper network. The experimental results are shown in the Results and demonstrate that YOLOv3 performed better than Faster R-CNN in this study.

### 3.9. PointNet

The 3D point cloud is a geometric data structure with three characteristics, described as follows:

(1) Disorder: The point cloud data are insensitive to the order of data.
(2) Space relationship: An object is usually composed of a certain number of point clouds in a specific space, where spatial relationships exist among these point clouds.
(3) Invariance: Point cloud data are invariant to certain spatial transformations, such as rotation and translation.

Because point cloud characteristics differ based on image data, existing frameworks for image classification cannot be applied to point clouds. Many studies have converted point cloud data into voxel grids [18,39] or images. Regarding voxel grids, point cloud data are divided into several cubes. Voxels exist with spatial dependencies. Additionally, 3D convolution and other methods are used to process these voxels. The accuracy of the method depends on the fineness of segmentation in 3D space. Notably, 3D convolution computation is complex and may represent a large computation load. However, images cannot be used to directly process 3D point cloud data because of the lack of distance information. Therefore, point cloud data are projected onto a 2D plane to provide a front or bird’s eye view of the data. This method can dramatically reduce the computation time. However, the methods mentioned above may lead to changes in the characteristics of the raw point cloud data and cause unnecessary data loss. To address this issue, a deep learning framework, PointNet [4], was proposed to directly process 3D point cloud data. 

Using PointNet, the input is the point cloud data (N*3), where N is the number of point clouds, and “3” represents the three coordinates: x, y, and z. First, the input data passes through a T-Net, which is used to learn the transformation matrices for data alignment. It normalizes the changes, such as rotation and translation of the point cloud. The input is raw point cloud data, and the output is a 3 × 3 matrix. This step can improve the model’s invariance to a specific spatial transformation. Second, after extracting the feature using a multi-layer perceptron (MLP), another T-Net is used to align the space feature. Third, max pooling is used to obtain the final global feature. For classification, the global feature can be used to classify an object by using an MLP. Thus, PointNet can use a sparse set of key points to describe the point cloud shape.

### 3.10. PointConv

PointNet++ [5], PointCNN [40], and SO-Net [41] are based on PointNet. They are one-dimensional convolution networks based on the multi-layer perceptron. The key structure in PointNet and PointNet++ is max pooling. It can preserve the strongest activation in local or global areas, but it may lose some useful detailed information. In PointCNN, an x-transform is learned in order to weight and replace the input features of a point. Unfortunately, it cannot realize permutation invariance. Instead, PointConv uses a new method to replace the previous PointNet structure, where the convolution is applied to a non-uniformly sampled 3D point cloud dataset.

For a d-dimensional vector x, the convolution is defined as follows:

(11) $$\left(f*g\right)\left(x\right)=\iint_{\tau \in {\mathbb{R}}^{d}}f\left(\tau \right)g\left(x+\tau \right)d\tau$$

For 2D images, the vector can be interpreted as a 2D discrete function and is presented as grid-shaped matrices. In the CNN, some convolution kernels are chosen to operate in a local region. The relative positions of different pixels are fixed in each local region because of the grid structure of the images. A 2D filter can then be discretized into a set of weights that act on the local region, and this set is called a convolution kernel. 

A 3D point cloud is a set of 3D points, where each point contains position information. The coordinates of points $p=\left(x,y,z\right)\in {\mathbb{R}}^{3}$ in the set are disordered. A natural grid structure does not exist, and thus points in the set are not located on a fixed grid. The traditional 2D CNN cannot be directly applied to a 3D point cloud. Therefore, the continuous version of the 3D convolution is as follows:

(12) $$Conv{\left(W,F\right)}_{xyz}={∭}_{\left(\delta_{x},\delta_{y},\delta_{z}\right)\epsilon G}W\left(\delta_{x},\delta_{y},\delta_{z}\right)F\left(x+\delta_{x},y+\delta_{y},z+\delta_{z}\right)d\delta_{x}\delta_{y}\delta_{z}$$

A point cloud, considered as a non-uniform sample in a continuous space ${\mathbb{R}}^{3}$. $\left(\delta_{x},\delta_{y},\delta_{z}\right),$ can occupy any position in a local region G. $F\left(x+\delta_{x},y+\delta_{y},z+\delta_{z}\right)$ is the feature of a point in the local region G centered around a point, where p=(x,y,z). Based on (12), PointConv is defined as follows:

(13) $$\begin{array}{l} PointConv{\left(S,W,F\right)}_{xyz}=\\ \;\sum_{\left(\delta_{x},\delta_{y},\delta_{z}\right)\epsilon G}S\left(\delta_{x},\delta_{y},\delta_{z}\right)W\left(\delta_{x},\delta_{y},\delta_{z}\right)F\left(x+\delta_{x},y+\delta_{y},z+\delta_{z}\right)d\delta_{x}\delta_{y}\delta_{z} \end{array}$$

where $S\left(\delta_{x},\delta_{y},\delta_{z}\right)$ is the inverse density at point $\left(\delta_{x},\delta_{y},\delta_{z}\right).$ $S\left(\delta_{x},\delta_{y},\delta_{z}\right)$ is necessary because point cloud sampling can be highly non-uniform. If the points are dense in a local region, the information exchanged among them is relatively low. The 3D coordinates $\left(\delta_{x},\delta_{y},\delta_{z}\right)$ are used to approximate the weight function $W\left(\delta_{x},\delta_{y},\delta_{z}\right)$ used by an MLP. Kernel density estimation and a non-linear transform, which are implemented by the MLP, are used to approximate the inverse density function $S\left(\delta_{x},\delta_{y},\delta_{z}\right)$. The density is used as the input to a 1D non-linear MLP. The non-linear transform allows the network to adaptively decide whether density estimation must be used. The weights in the MLP are shared among all points to ensure permutation invariance. In a native version of PointConv, random-access memory will be dramatically depleted and processing is inefficient. Unlike [42], a novel reformulation is used in PointConv to reduce this problem, namely using matrix multiplication and 2D convolution. This method can not only operate under parallel computing using a graphics processing unit (GPU), but can also be reproduced on mainstream deep learning frameworks.

## 4. Results

The experimental field located at the fishing port in Kaohsiung, Taiwan. The water depth was about 5 m. There were many abandoned tires on the seabed. To identify a tire underwater, which was the aim of this study, a sensor was used to obtain precise data. To this end, the BV5000 3D acoustic sensor was used to collect a point cloud underwater. The BV5000 was deployed at different locations to collect data by different views. The high-resolution data thus obtained clearly depicted the underwater environment; however, there were many redundant points present in the raw data. The raw data were therefore pre-processed for noise removal. After pre-processing, the remaining data were converted to two data types, which enabled the data to be analyzed from two different dimensional aspects. The first type was 2D data results used to create an image-based type. The Faster R-CNN and YOLOv3 neural networks were used to train the models to detect tires in the 2D images. The second type depicted 3D data results as a point cloud-based type. PointNet and PointConv neural networks were used to train the models to classify the data from the point cloud.

### 4.1. Two-Dimensional Image Detection

**Figure 6:**
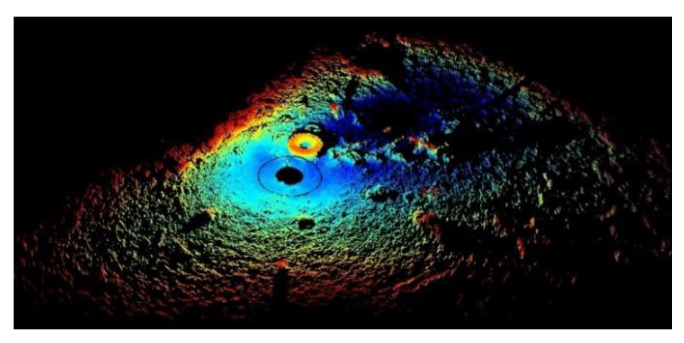
3D results after choosing the ROI.

**Figure 7:**
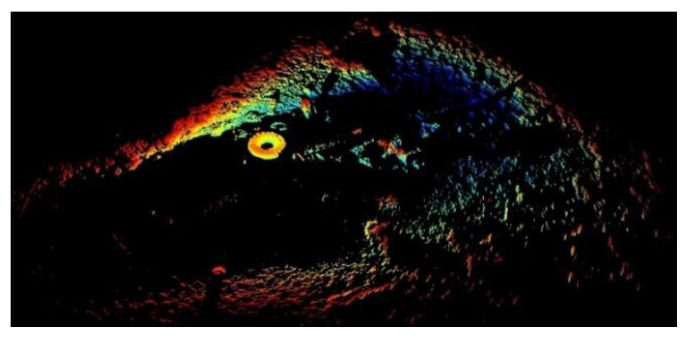
3D results after removing the seabed.

**Figure 8:**
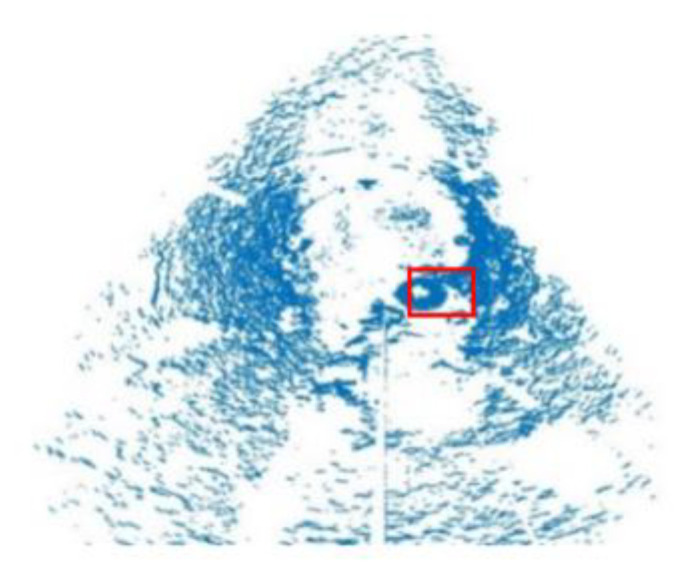
2D bird’s eye view of the point cloud.

In this study, 3D mechanical scanning sonar was used to obtain 3D data. The 3D data were compressed to 2D data to obtain a flat image of the data. First, the boundary points in the raw data was removed. The results are shown in Figure 6. Second, the RANSAC algorithm was used to remove the seabed. The results are shown in Figure 7. Third, the height was set to zero to obtain a bird’s eye (2D) view of the environment, and the outcome was shown in Figure 8. Because the height value was set to zero, the color of all points was the same. Compared to 3D data, 2D data provided a visualization results and did not need great computing ability. 

Although there are point clouds representing several objects on the seabed, as depicted in Figure 8, the tire is clearly visible (red rectangle). The output image data were labeled to mark the coordinates of the tire. The training data consisted of 400 images and the test data consisted of 176 images. 

**Figure 9:**
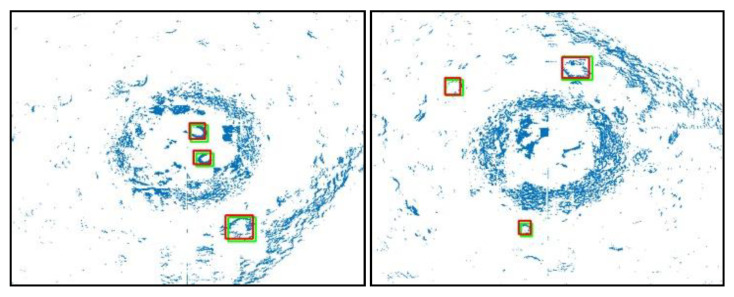
Results for 2D detection.

Figure 9 depicts the detection results for YOLOv3. In both images, the green rectangles denote the ground truth, while the red rectangles denote bounding boxes. The models can correctly detect the tires from the 2D data.

**Figure 10:**
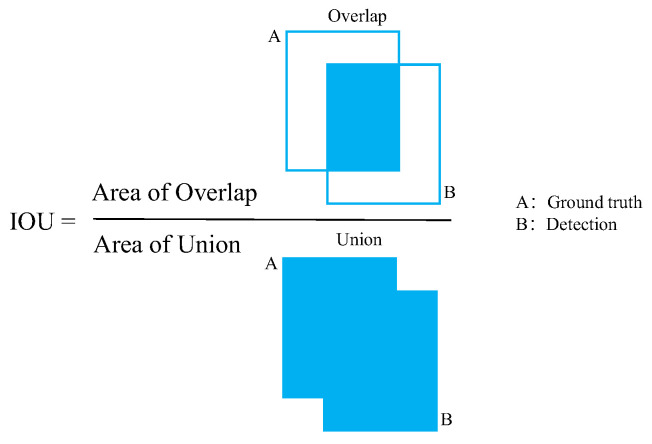
Intersection Over the Union.

A confusion matrix was applied to evaluate the performance of the models, as presented in Table 2. The intersection over union (IOU) was calculated to analyze the results. The IOU threshold was set as 0.5 based on the experimental results. In this experiment, if the IOU threshold was over 0.5, some incorrect objects would be identified as tires. If the IOU value was under 0.5, many small tires would not be found because low deviations in coordination would cause a dramatic decrease in the IOU. A schematic of the estimated IOU is shown in Figure 10. The blue square (the numerator) denotes the overlap between the two rectangles, and the denominator is the union of the two rectangles. The ground truth was manually labeled and used to verify the detection results of the models. The decision method regarding whether the object is a tire is as follows:

(1) Every bounding box from the model was used to calculate the IOU of the ground truth. If the value was larger than the IOU threshold, it was considered true, and vice versa. If more than one bounding box was true, the bounding box with the highest IOU was set as true, and the others were set as false.
(2) The bounding box prediction scores were sorted in the order of highest to lowest. If a bounding box was true, it was considered to be a true positive. Otherwise, it was considered to be a false positive. The precision and recall values were estimated using (15) and (16).
(3) If the object existed in the ground truth, but the model did not detect it, it was regarded as a false negative.
(4) If the model detected the object but the object did not exist in the ground truth, it was regarded as a false positive.
(5) Every tire in the image would be detected by the steps mentioned above and obtained the results. This method can precisely analyze whether the model can obtain correct results for every tire.

As the confusion matrix only compiles statistics, it is difficult to fully evaluate the performance of the models. To further analyze the detection results, the confusion matrix is extended using four indicators based on the basic statistical results. These are called secondary indicators. They include accuracy, precision, sensitivity, and specificity, and are defined as follows:

(14) $$Accuracy=\frac{TP+TN}{TP+TN+FP+FN}$$

(15) $$Precision=\frac{TP}{TP+FP}$$

(16) $$Sensitivity=Recall=\frac{TP}{Ground\;truth}$$

(17) $$Specificity=\frac{TN}{TN+FP}$$

The four secondary indicators can generate the data in a confusion matrix as a ratio ranging from 0 to 1. It is easy to perform standardized measurements in this case. A third indicator, the *F*1 score, can be determined from the four indicators. It combines the outputs of precision and recall. The model is more stable if the *F*1 score is higher.

(18) $$F1\;score=\frac{2*precision*recall}{precision+recall}$$

With an IOU threshold of 0.5, the secondary indicators and the *F*1 score results are shown in Table 3. It was found that YOLOv3 performs better than Faster R-CNN.

**Figure 11:**
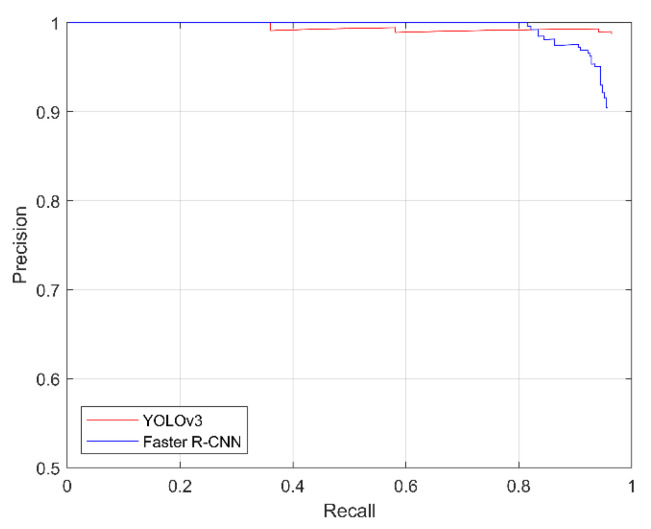
Precision-recall curves for the YOLOv3 and Faster R-CNN neural networks.

To investigate the variety of each bounding box, every bounding box prediction score was sorted from highest to lowest to calculate the precision and recall values. The IOU threshold is 0.5. Based on the different prediction scores, the precision-recall curve (PR-Curve) could be drawn. The results are shown in Figure 11. As the prediction score threshold decreased, the precision of Faster R-CNN decreased more quickly than that of YOLOv3. Due to included more incorrect bounding boxes with low predicted scores deteriorate the performance of detection in Faster R-CNN model. If the prediction score threshold decreased, the number of false positives would increase and caused the decrease of precision.

As opposed to a graph, specific individual values can intuitively demonstrate the performance of the models. Therefore, the average precision (AP) was calculated. As indicated by formula (19), the AP is the area of the PR curve, where P is the precision and r is the recall. The AP results are shown in Table 3. It can be seen that the AP values of both models are similar, but that of YOLOv3 is slightly higher:

(19) $$\mathrm{AP}=\int_{0}^{1}P\left(r\right)\mathrm{dr}$$

### 4.2. Three-Dimensional Point Cloud Classification

**Figure 12:**
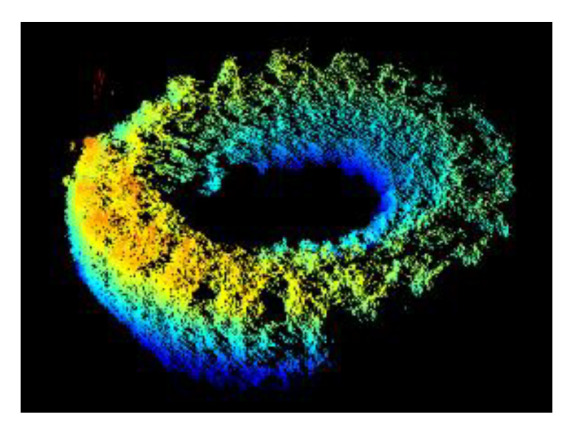
After removing the tire from the entire point cloud.

Point cloud data are being increasingly used for object detection. Unlike 2D image data, 3D point cloud data can retain information such as the shape and size of an object. Many studies involve transferring point clouds into voxels or a graph; however, this may lead to loss of detail during data conversion. In this study, the point cloud of the tire was extracted from the raw data without performing any data conversion to ensure that no information was lost. First, the boundary points in the raw data was removed. Second, the RANSAC algorithm was used to remove the seabed. Third, another ROI was used to extract the tire from the data. The complete shape of the tire is shown in Figure 12. Fourth, the point cloud was rotated in 5° increments to augment the data. Fifth, the data were then transformed to hdf5 format.

To increase data diversity, the ModelNet40 dataset and a tire dataset were integrated during model training and used to evaluate the efficiency of the models. The ModelNet40 dataset includes 40 classes, so that the total number of classes in the point cloud data was increased from 40 to 41. These 41 classes were used to train the models. The number of training data was 10,704, while that of the testing data was 2900. PointNet and PointConv were the neural networks used in the 3D study. The models were used to evaluate two types of datasets, one of which was ModelNet40 with 40 classes, and the second dataset was ModelNet40 data mixed with tire data. The results are presented in Table 4. In Table 4, the accuracy of the original PointNet model in ModelNet40 was 89.2%, while the new PointNet model trained using 41 types of objects was 88.2% on ModelNet40 and 87.4% on a mixture of ModelNet40 and tire data. The accuracy of the original PointConv model in ModelNet40 was 92.5%, and the new PointConv model trained using 41 types of objects was 91.5% on ModelNet40 and was 93% on a mixture of ModelNet40 and tire data. Although the accuracy of our models may have decreased by 1% for the ModelNet40 dataset, the results are comparable to those obtained in previous studies. The decrease in accuracy is believed to be caused by the combination of the ModelNet40 dataset and the point cloud data of the tires. The tire point cloud data slightly influenced the original classification of the 40 classes. However, both neural networks achieved 100% accuracy when ModelNet40 and tire data were combined. 

Based on the results of the 3D data classification, this study concludes that point cloud classification can be realized on land and can be extended to underwater applications using acoustic sensors. Furthermore, the use of the pre-processing steps ensured better classification of underwater objects.

## 5. Conclusions

This research extended 2D detection and 3D deep learning applications from land to underwater object classification. In the experiment involving 2D data, to verify that our model can deal well with different field of views of the tire, the tripod was moved to get data from the same tire. The ROI was set such that any redundant point clouds were removed. The RANSAC algorithm was then applied to remove the seabed. A 3D point cloud was compressed to obtain a 2D image of the data. Faster R-CNN and YOLOv3 were used to detect any tires in the image. The results indicate that the models can correctly detect tires from different field of views and YOLOv3 obtained better results than Faster R-CNN. In the 3D data experiment, the same initial pre-processing steps were followed as in the case of the experiment with 2D data. However, to obtain the complete point cloud of a tire, another ROI was set to extract the tire from the entire point cloud. PointNet and PointConv were then trained for detection, and the results indicate that the models obtained excellent classification results when considering a 3D point cloud. The accuracy of the PointConv model was better than that of the PointNet model.

The proposed methodology extends 3D point cloud application from ground to underwater. Currently we only collect one kind of target to approach our methods. In the future, we want to collect more underwater targets like bikes, anchors or other interesting objects to expand underwater point cloud dataset. This cloud be useful to help detect lost objects underwater. 

## Figures and Tables

**Table 1:** Comparison of the three sonar types.

	Side-Scan	Multi-Beam	BV5000
Installation	Mount under the vehicle	Mount under the vehicle	Placed on the seabed
Other sensor requirements	GPS	GPS, IMU	No
Measurement method	Sailing back and forth	Sailing back and forth	Multiple measurement station
Measurement range	150 m on both sides	User-selected	30 m in radius
Resolution	5 cm	10 cm	1 cm
Application	Target search	Submarine geomorphology survey	Inspect dock structure
Difficulty of operation	Pay attention to vehicle speed and distance from the sea floor	Inappropriate for shallow water because of post-processing difficulties	Easy to operate

**Table 2:** Confusion matrix.

Confusion Matrix		Ground truth	
		Tire	Not a Tire
**Prediction**	Tire	True Positive (TP)	False Positive (FP)
	Not a tire	False Negative (FN)	True Negative (TN)

**Table 3:** Comparison of the two models.

Model	Faster R-CNN	YOLOv3
Accuracy (%)	87.0	95.8
Precision (%)	90.5	98.7
Recall (%)	95.8	96.4
*F*1 (%)	93.0	97.5
AP	0.954	0.96

**Table 4:** Classification results using Modelnet40 and tire data.

Method	Class	Only ModelNet40	ModelNet40 & Tire
PointNet	40	89.2	-
PointConv	40	92.5	-
Expanded PointNet	41	88.1	87.4
Expanded PointConv	41	91.5	93
